# Adherence of trials of operative intervention to the CONSORT statement

**DOI:** 10.1308/003588414X13824511649418

**Published:** 2014-01

**Authors:** ER Carthy, R Agha, R Gray, M Sullivan, DG Altman, AN Gordon-Weeks

**Affiliations:** Imperial College London,UK; National Institute for Health and Care Excellence,UK

This paper by Gray *et al* highlighted the inadequacy in the reporting of non-pharmacological randomised controlled trials. This is consistent with previous work by our group,^1,2^ which has demonstrated the huge need for improvement with regard to the methodological reporting of surgical trials.

Gray *et al* quote a statistically significant improvement in adherence to the Consolidated Standards of Reporting Trials statement for non-pharmacological treatment (CON-SORT-NPT) of 3.95 points (*p*<0.001). This is indeed slightly higher than of previous work^2^ but it is still indicative of poor compliance across multiple surgical specialties, with minimal improvement over the last decade.

The guidance set out by CONSORT-NPT needs to be ‘hard-wired’ into the submission process across all surgical publications. Furthermore, compliance needs to be emphasised and adhered to by all stakeholders, be they authors, reviewers, publishers, editors, funders or readers. Regular checks and consistency in quality control is paramount to ensuring the highest possible standards of reporting.
